# A Pleasant Mouth Disinfectant

**Published:** 1866-09

**Authors:** 


A Pleasant Mouth Disinfectant:—Hypermangate of potassa and hyperoxydate of barium, of each twenty-four grains, to be rubbed up into a mass, with sugar and glycerin, and divided into 144 lozenges. Every ill-smelling mouth will become by their use perfectly odorless.—*Medical Record.*

Gunpowder Marks.—Smear the scorched places with glycerin, by means of a feather, then apply cotton wadding; lastly cover with oil silk. In one case the discoloration was very great, the patient looking more like a mummy than a living being. It entirely subsided in a month by the above treatment.—*London Lancet.*

